# Rheological Properties, Compatibility, and Storage Stability of SBS Latex-Modified Asphalt

**DOI:** 10.3390/ma12223683

**Published:** 2019-11-08

**Authors:** Shisong Ren, Xueyan Liu, Weiyu Fan, Haopeng Wang, Sandra Erkens

**Affiliations:** 1 Section of Pavement Engineering, Faculty of Civil Engineering and Geosciences, Delft University of Technology, Stevinweg 1, 2628 CN Delft, The Netherlandsx.liu@tudelft.nl (X.L.); S.M.J.G.Erkens@tudelft.nl (S.E.); 2 State Key Laboratory of Heavy Oil Processing, China University of Petroleum, Qingdao 266580, Shandong, China

**Keywords:** SBS-modified asphalt, SBS latex, rheological properties, compatibility, storage stability

## Abstract

A styrene-butadiene-styrene (SBS) latex modifier can be used for asphalt modification due to the fact of its energy-saving, construction convenience, and economic advantages. The objective of this study was to investigate the influence of asphalt type and SBS latex dosage on the rheological properties, compatibility, and storage stability of asphalt through temperature and frequency sweep, steady-state flow, multiple stress creep and recovery (MSCR) tests, Cole-Cole plots and thermal storage tests. The results indicated that high SBS latex content is beneficial for improving anti-rutting, anti-fatigue, viscous flow resistance, and elastic recovery abilities of modified asphalt. The chemical composition of asphalt had a significant effect on the properties of the SBS latex-modified asphalt. High asphaltenes and low resins were favorable to enhancing anti-rutting and recovery properties but weakened the anti-fatigue, compatibility, and storage stability of modified asphalt. Furthermore, compared to SBS particle-modified asphalt, SBS latex-modified asphalt had greater rutting and fatigue resistance. However, SBS latex-modified asphalt had some disadvantages in compatibility and storage stability. Comprehensively considering the balance of viscoelastic properties, compatibility, and storage stability of SBS latex-modified asphalt, the mixing dosage of SBS latex modifier is recommended at 4.0 wt% which could feasibly replace SBS particle in asphalt modification.

## 1. Introduction

Asphalt is always used as binder material for road construction, and it is obtained from petroleum refining processes [1]. In terms of chemical composition, asphalt is composed of saturates, aromatics, resins, and asphaltenes. The main elements of bitumen is C, H, S, N, and O which is similar to petroleum products. Currently, with the increase in traffic loading and temperature conditions, asphalt roads are easily damaged including rutting, cracking, flacking, etc. [2,3]. Therefore, it is urgently needed to improve the performance of asphalt pavement and prolong its service life.

It is clear that base asphalt has many disadvantages, such as high-temperature flow, low-temperature cracking, and temperature susceptibility, that make it incapable of meeting the performance requirements of high-grade pavement [4]. Thus, in order to guarantee the pavement properties of an asphalt mixture under complex environments, additives have been used to modify asphalt. The main modifiers for asphalt binder include styrene-butadiene-styrene (SBS), crumb rubber (CR), polythene (PE), ethylene-vinyl acetate (EVA), styrene-butadiene rubber (SBR), polyphosphoric acid (PPA), gilsonite, and other nanomaterials [1,5,6]. According to previous studies [2,4], the addition of a polymer modifier can effectively improve the high-temperature rutting and low-temperature cracking resistance of asphalt and prolong the service life of the pavement. Zhang et al. [7] studied the effect of SBS on the rheological and aging properties of asphalt, and the results showed that SBS could remarkably enhance the viscoelastic performance and anti-aging capacity of asphalt. Meanwhile, Liang et al. [8] investigated the influence of sulfur and SBS type on both the properties and mechanism of SBS-modified asphalt, showing that there existed a chemical reaction between the SBS copolymer and the base asphalt. The SBS polymer molecule in asphalt can crosslink which results in the formation of a three-dimensional polymer network structure. This is why SBS-modified asphalt possesses better high- and low-temperature properties.

On the other hand, it is obvious that SBS-modified asphalt has disadvantages in terms of compatibility and storage stability [9]. Another problem is that modified asphalt with high-content SBS has poor workability and low economic efficiency which further limits the application of SBS copolymer [10,11]. Recently, researchers proposed that other additives can be added into SBS-modified asphalt to prepare complex modified asphalt which can alleviate the stability issue and further improve the pavement performance of SBS-modified asphalt. Qian et al. [12] investigated and concluded that CR/SBS composite-modified asphalt produced via a high-cured method had better performance, and it was a cost-effective approach to producing asphalt binder. In addition, Li et al. [13] prepared a modified heavy calcium carbonate and SBS composite-modified asphalt and found that the addition of calcium carbonate could strengthen the modulus and better enhance the viscoelastic properties than pristine SBS-modified asphalt. Importantly, SBS-modified asphalt is always manufactured by the asphalt industry because of the high-speed shear process used, and then it is transported to the construction site where asphalt binder and aggregate are mixed and compacted. Thus, separation between asphalt and SBS could easily happen during thermal storage and transportation [2,14]. Moreover, the preparation process for SBS-modified asphalt is complicated, including high-speed shear and mixing, and is energy intensive and costly.

Both the storage stability and the compatibility of SBS-modified asphalt are important to its engineering application and performance in asphalt roads. Clearly, a strong phase separation of the SBS copolymer from bitumen during storage and transportation is not expected [15]. Many researchers have studied the effects of SBS dosage on the compatibility and storage stability of modified asphalt. Lu et al. [16] investigated the compatibility and storage stability of SBS-modified bitumen using fluorescence microscopy and dynamic mechanical analysis. The results showed that the storage stability of modified asphalt decreased with an increase in SBS content; in addition, the degree of SBS dispersion in bitumen influenced the storage stability and rheological properties of modified binders as well. Meanwhile, Fu et al. [17] added SBS-g-M grafted with vinyl monomer into SBS-modified asphalt and found that the compatibility of the SBS-modified asphalt improved significantly. Therefore, the content of SBS modifier needs to be controlled to prevent the modified asphalt from phase separation.

**Figure 1:**
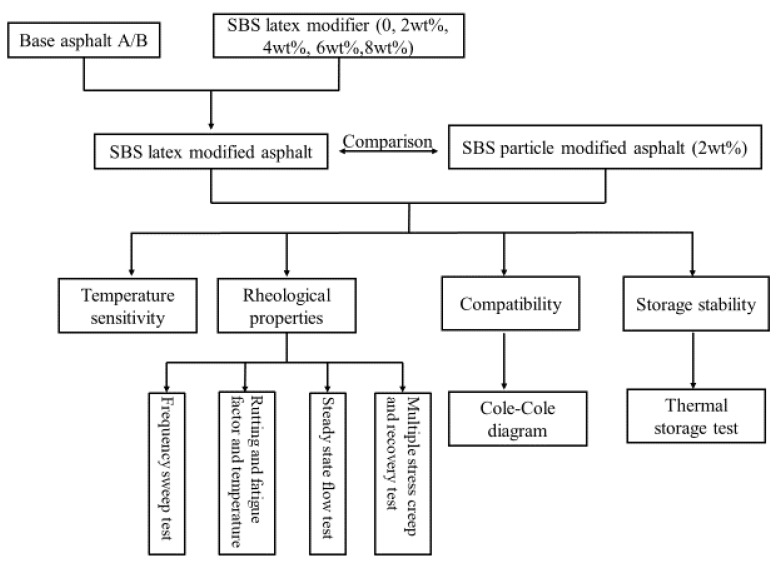
Flow chart of the experimental projects.

The objective of this study was to explore the possibility of using an SBS latex modifier in asphalt modification and to investigate the effects of SBS latex dosage and asphalt components on the rheological properties, compatibility, and storage stability of modified asphalt. The experimental work in this study is shown in Figure 1 and described in the subsequent sections.

## 2. Materials and Methods

### 2.1. Raw Materials

In this paper, two base asphalts (PetroChina Fuel Oil Co. Ltd., Beijing, China) (coded as A and B) with a 60/80 penetration grade were used. The conventional properties and chemical compositions of these two base asphalts are displayed in Table 1. It is clear that, although the two asphalts have a similar penetration grade, their components have significant differences. The SBS latex modifier was provided by Shandong Dashan Road&Bridge Engineering Co. Ltd., Shandong Province, China. The apparent condition of the SBS-latex modifier showed milky liquid. The SBS solid concentration in latex modifier was 33.55 wt%. The SBS latex is the product prior to the preparation of the SBS particle. Owing to the lack of concentration and prilling procedure, a large amount of solvent exists in SBS latex. Therefore, the preparation of SBS latex-modified asphalt can not only omit the solidification and off-solvent operational processes, but it can also be prepared with only the use of a mixing stirrer.

### 2.2. Preparation of SBS Latex-Modified Asphalt

**Figure 2:**
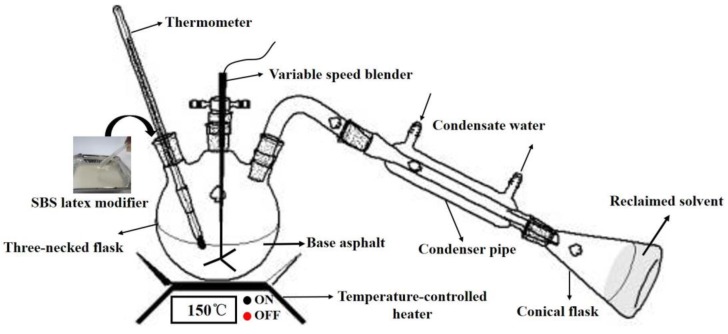
The preparation process of styrene-butadiene-styrene (SBS) latex-modified asphalt.

In this study, the preparation process of the SBS-latex-modified asphalt is shown in Figure 2. This process can be separated into three parts: the mixing section, condensation part, and recovery device. The dosage of the SBS latex modifier was determined in accordance with previous studies [22,23]. Firstly, the base asphalt was preheated in a temperature-controlled oven to a flow state. Then, it was poured into a three-necked flask which was heated in a cylindrical container to 150 °C and mixed with a certain amount of SBS latex modifier (0, 2 wt%, 4 wt%, 6 wt%, and 8 wt% of neat asphalt) with a speed of 1000 rpm for 30 min using a variable-speed blender. After that, 0.2 wt% sulfur powder was added into the above blend which was then mixed at 1000 rpm and 150 °C for 30 min. For simplicity, the modified asphalt A (B) containing various SBS latex dosages are abbreviated as A0 (B0), A2 (B2), A4 (B4), A6 (B6), and A8 (B8). Owing to the existence of solvent in SBS latex, the preparation device for SBS latex-modified asphalt was closed and equipped with a solvent condensation and recovery unit which can be seen in Figure 2. In order to evaluate and compare the performance grades of the SBS latex-modified asphalt, a conventional SBS particle-modified asphalt with the same SBS solid content was also prepared. The base asphalt was preheated to 175 °C and then the SBS particle modifier (the same solid content with 6 wt% SBS latex) was added and was sheared at 4000 rpm for 30 min using a high shear machine. Then, 0.2 wt% sulfur powder was added into the above blend and mixed at 1000 rpm and 150 °C for 30 min. Finally, the conventional SBS particle-modified asphalt was obtained and named ASBS (BSBS).

### 2.3. Test Methods

In this study, SBS latex-modified asphalts with various SBS latex dosages were prepared via distilling and condensing device. Then, dynamic shear rheometer (DSR) (Anton Par, Graz, Austrian) was conducted to analyze the viscoelastic properties of the modified asphalts. In addition, Cole-Cole diagrams and thermal storage tests were performed to evaluate the compatibility and storage stability of the SBS latex-modified asphalts, respectively. Finally, the performance of the SBS latex-modified asphalt was compared with the conventional SBS particle-modified asphalt, and the optimum content of SBS latex modifier was obtained.

Rheological characterization of the asphalt samples was performed using the DSR device [24]. Frequency sweep tests were conducted at 60 °C with the frequency increasing from 0.1 rad/s to 100 rad/s. In addition, temperature sweep tests were performed at 10 rad/s from 48 °C to 84 °C with a parallel plate geometry of 25 mm and a gap setting of 1 mm to measure the moduli and rutting factor of the asphalts. Meanwhile, fatigue factors were also obtained by temperature sweep tests at 10 rad/s with the testing temperature increasing from 10 °C to 50 °C. To guarantee the linear viscoelastic ranges of the asphalt, train sweep tests were conducted to determine the loaded strain before these two tests.

In addition, a steady-state flow method was performed to obtain the influence of SBS latex dosage on the zero-shear viscosity (ZSV) and deformation resistance abilities of modified asphalt [20]. The shear rate regions were chosen from 10^−3^ s^−1^ to 10^2^ s^−1^. Furthermore, multiple stress creep and recovery tests (MSCR), employed under two loading levels of 0.1 KPa and 3.2 KPa, were conducted at 60 °C. For both the loading levels, the asphalt samples underwent ten cycles of one-second creep and nine seconds recovery [25,26]. The applied parallel plates’ geometry and gap were determined according to the relevant literatures [27,28,29].

## 3. Results and Discussion

### 3.1. Frequency Sweep Tests

**Figure 3:**
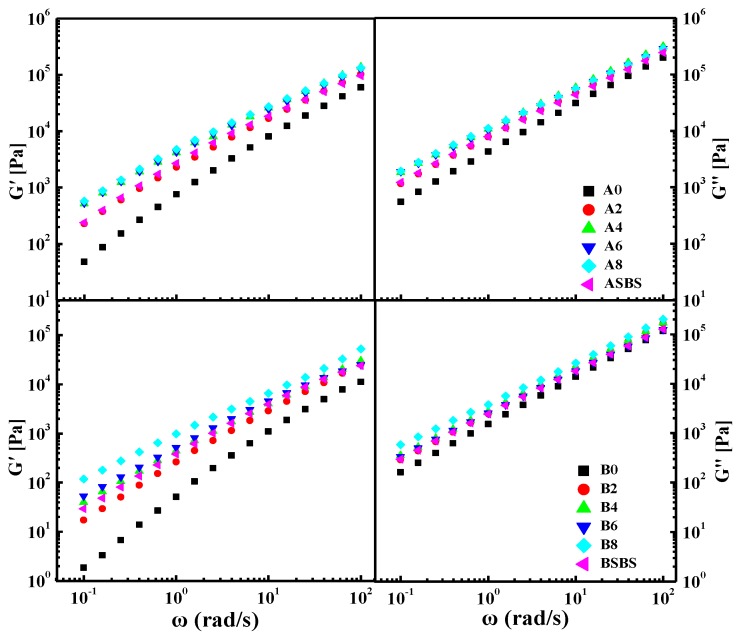
Effects of loading frequency on the storage and loss modulus of the base and the SBS latex-modified asphalts.

It is clear that road performance depends, to some extent, on the viscoelastic and mechanical properties of asphalt binder [30,31]. Characterization of the linear viscoelastic behavior of the asphalt binder was performed to evaluate the effect of the SBS latex dosage on the rheology of the modified asphalts. Asphalt is a viscoelastic material that behaves very differently with various temperature and loading times. Therefore, a frequency sweep test was performed in the frequency region of 0.1–100 rad/s at 60 °C, and the experimental results are shown in Figure 3.

From Figure 3, clear increasing trends in the storage modulus G’ and loss modulus G” can be seen with the frequency increasing gradually. It is well known that storage modulus G’ represents the energy recovery and elastic section of asphalt, while loss modulus G” shows the energy loss and viscous part. However, the increasing trends of the two moduli were very heterogeneous. With regard to all asphalt samples, storage modulus G’ showed lower values than loss modulus G” in the whole testing frequency region, indicating that the viscoelastic performance of the base and modified asphalts were primarily dominated by viscous properties.

This phenomenon was more obvious in the case of neat asphalt which is attributed to its poor viscoelastic properties at high temperature. Meanwhile, the gap between G’ and G” became smaller as the frequency declined. After adding SBS latex, the G’ and G” values of the modified asphalt both underwent an increasing trend compared with the neat asphalt. With the increase in the SBS latex dosage, the G’ and G” values of asphalt samples both continued to increased, while the increasing trend weakened when the SBS latex content exceeds 6 wt%. The results also reveal that adding SBS latex increased the proportion of elastic components in the binder, which contributes to improving the stiffness of asphalt at high temperature.

Furthermore, asphalt composition also has a remarkable influence on the modulus and stiffness of SBS latex-modified asphalt. Both the storage and loss modulus of asphalt A were higher than that of asphalt B with the same SBS latex concentration; this is attributed to the differences in asphalt composition. This phenomenon suggests that there was a stronger reaction between the SBS latex modifier and base asphalt A with a higher asphaltenes content. As a consequence, asphalt type and composition are very crucial for SBS latex modification, and a relatively high asphaltenes dosage is beneficial to obtaining a satisfactory modification result. Additionally, the SBS latex-modified asphalt had better moduli than that of the conventional SBS particle-modified asphalt when the SBS concentration was the same, indicating that the SBS latex-modified asphalt possessed better deformation resistance performance. In addition, the moduli difference between the SBS latex-modified asphalt and the conventional SBS particle-modified asphalt was more distinct for asphalt A.

### 3.2. Temperature Sweep Tests

**Figure 4:**
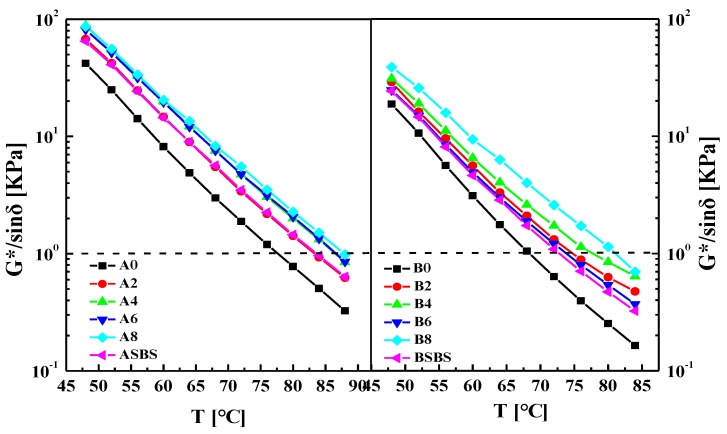
Effects of temperature on the rutting factor of the base and SBS-latex-modified asphalts.

It is well known that rutting resistance factor G*/sinδ is an important index to evaluate the high-temperature stability of asphalt. The rutting resistance factor G*/sinδ of asphalt was obtained by temperature sweep tests with the temperature increasing from 48 °C to 84 °C and with a frequency of 10 rad/s. The rutting resistance factors G*/sinδ of the base and modified asphalts with various SBS latex dosages are presented in Figure 4. There was an obvious linear relationship between the testing temperature and rutting resistance factors of the asphalts. In order to evaluate the impact of SBS latex content on the anti-rutting properties of the modified asphalts, the linear fitting formula was used and the failure temperatures of the asphalts were calculated when the rutting resistance factor G*/sinδ was equal to 1.0 KPa.

Table 2 shows the failure temperatures of the base and modified asphalts. It can be seen that the addition of SBS latex increased the failure temperature of the base asphalt dramatically, indicating SBS latex can improve the anti-rutting ability of asphalt. However, the SBS latex dosage had a different effect on the improvement of the failure temperature of the asphalts. For asphalt A, the failure temperature increased by 7.01%, 10.74%, 10.79%, and 12.25% when the SBS latex content was 2 wt%, 4 wt%, 6 wt%, and 8 wt%, respectively, while the failure temperature of asphalt B increased by 9.15%, 14.28%, 15.27%, and 18.42%. Clearly, the greater influence of the SBS latex concentration on asphalt B over asphalt A is obvious. Meanwhile, the degree of influence became smaller when the SBS latex dosage exceeded 4 wt%.

In addition, the failure temperature of the SBS latex-modified asphalt was higher than that of the SBS particle-modified asphalt with the same SBS solid content. That is to say, SBS latex-modified asphalt had a better anti-rutting ability than the SBS particle-modified asphalt which is the main binder used in pavement construction. Moreover, for asphalt A, the failure temperature of the SBS latex-modified asphalt was 2.67 °C larger than that of the SBS particle-modified asphalt, while the difference for asphalt B was 6.15 °C. Meanwhile, the failure temperature values of the modified asphalts A were all higher than that of asphalts B, showing that the SBS latex-modified asphalt A had greater rutting and deformation resistance because of the higher asphaltenes concentration.

**Figure 5:**
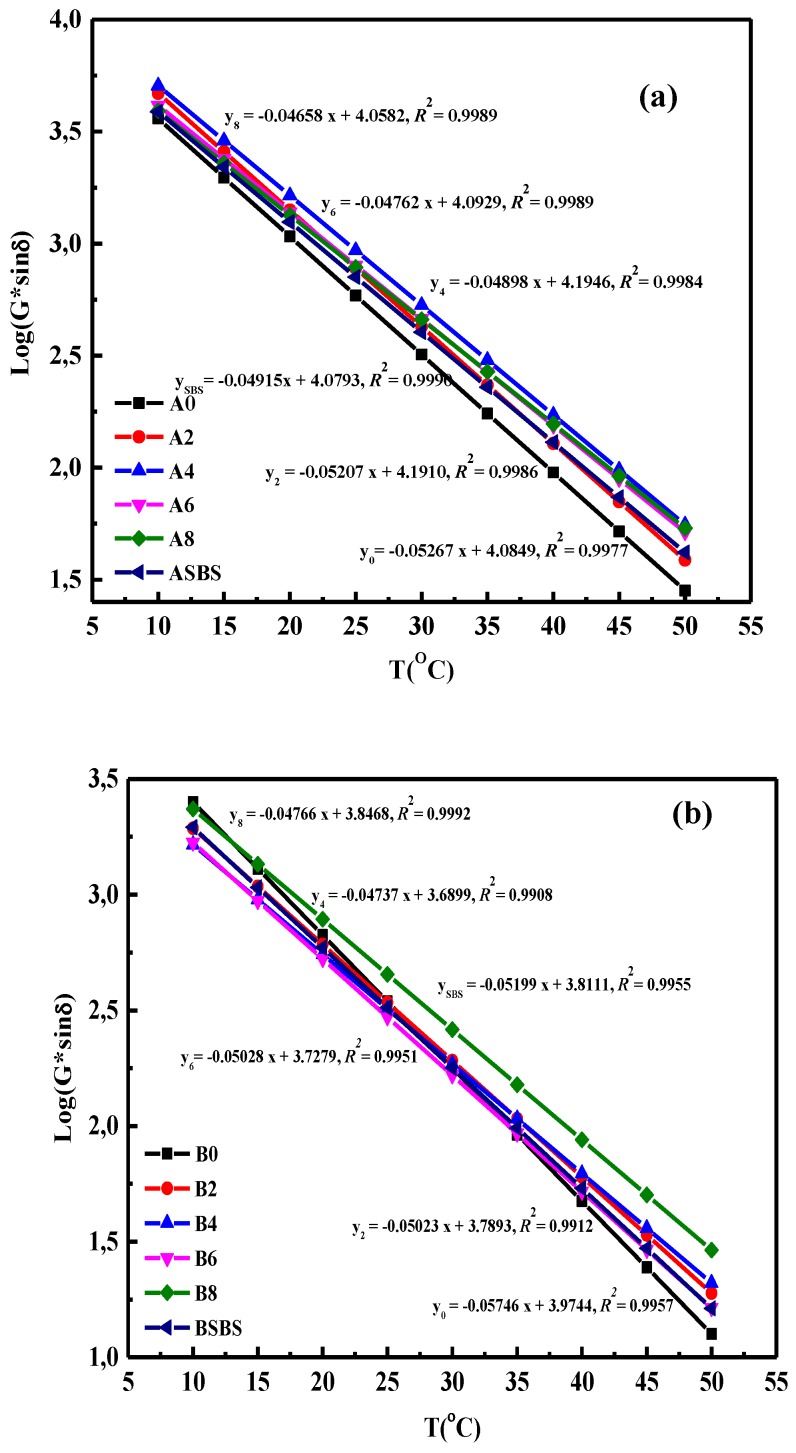
Effects of temperature on the fatigue factor of (**a**) the base and (**b**) the SBS latex-modified asphalts.

Fatigue cracking is another defect of asphalt pavement, thus the fatigue resistance factor is an important and essential index to evaluate the long-term pavement performance and service life of asphalt roads which has been receiving increasing attention [32,33]. In this study, temperature sweep tests were performed with temperature changes from 10 °C to 50 °C with 5 °C increments to investigate the effect of SBS latex dosage on the fatigue resistance of modified asphalt. The relationship between testing temperature and fatigue factors G*sinδ in SBS-latex-modified asphalt is plotted in Figure 5. It was found that the fatigue resistance factor of modified asphalt increased as the temperature gradually increased, illustrating that a high-temperature environment has an adverse effect on the fatigue resistance performance of SBS latex-modified asphalt.

In order to assess the fatigue property of modified asphalt intuitively, the ultimate fatigue temperature was calculated and used for analysis [34]. The relationship between the temperature and fatigue resistance of asphalt can be demonstrated by a linear formula, which is also shown in Figure 5. There was a clear linear relationship between the temperature and fatigue factor of asphalt, and the correlation coefficient values *R*^2^ were all more than 0.99. Besides, the fatigue temperature of of base and modified asphalts with various SBS latex concentrations are displayed in Table 2. The SBS latex dosage has great influence on the fatigue property of modified asphalt. The addition of SBS latex remarkably decreased the fatigue temperature of base asphalt, showing the positive effect of SBS latex on improving the fatigue resistance of asphalt. However, as SBS latex dosage increased, the fatigue temperature of modified asphalt first declined and then increased, indicating that a higher SBS latex dosage leads to a loss in fatigue resistance for SBS latex-modified asphalt, and that there exists an optimal SBS latex content to ensure the best fatigue resistance performance. Regardless of the asphalt components, the modified asphalt with an SBS latex content of 4 wt% had the lowest fatigue temperature and best fatigue resistance ability. That is to say, the optimal dosage of SBS latex in this study was 4 wt% when considering the anti-fatigue performance of modified asphalt.

Furthermore, asphalt type and chemical composition also affected the fatigue resistance of SBS-latex-modified asphalt dramatically [35,36]. It was found that, with the same SBS latex dosage, the fatigue temperature values of modified asphalts A were all higher than that of modified asphalts B, indicating that modified asphalts B had better fatigue resistance performance. This is due to the difference in asphalt composition, and the lower asphaltene concentration of asphalt B contributed to the improvement of fatigue resistance performance. It is interesting that the SBS latex-modified asphalt had a lower fatigue temperature than the SBS particle-modified asphalt, indicating that the SBS latex-modified asphalt had better fatigue resistance. Thus, in regard to the fatigue resistance properties, it is feasible and superior to use SBS latex as a modifier of asphalt to enhance its properties.

### 3.3. Steady-State Flow Tests

**Figure 6:**
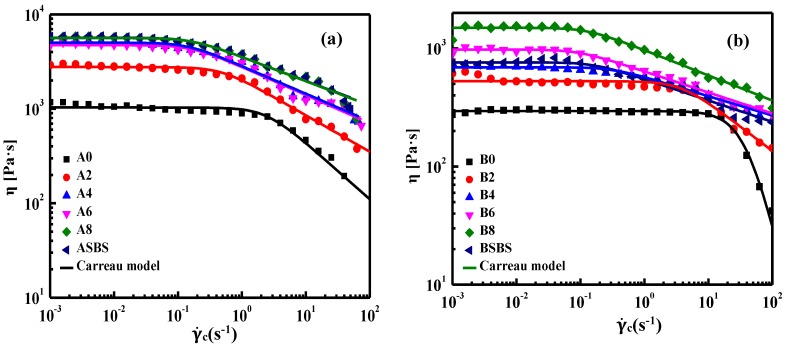
Flow curves of (**a**) the base and (**b**)SBS-latex-modified asphalts.

Characterization under large deformation always gives a very distinct viscoelastic response in comparison with those in small deformation, especially for polymer-modified asphalt [37,38]. Thus, the 60 °C flow behavior of asphalt was characterized by steady-state flow tests with the shear rate increasing from 10^−3^ s^−1^ to 10^2^ s^−1^. The flow curves of base and SBS-latex-modified asphalts are plotted in Figure 6. On the whole, the viscous flow behavior of asphalt shows a dependence on shear rate, which is extraordinarily distinct for modified asphalt. The shear viscosity of asphalt keeps constant when the shear rate is in the low range, representing the typical Newtonian fluid characteristic [39]. When the shear rate exceeds this value, the shear viscosity of the asphalt decreases remarkably which presents an apparent shear-thinning behavior. Neat asphalt has the widest Newtonian behavior region, which is shortened after adding SBS latex. The non-Newtonian fluid characteristics of asphalt with a higher SBS latex dosage were more obvious, which is attributed to the complicated entanglement between asphalt and polymer. On the other hand, the viscosity of the base asphalt was the lowest, increasing gradually with the increase in SBS latex content, indicating that adding SBS latex can improve the flow resistance of asphalt dramatically. Overall, base asphalt presented primarily Newtonian fluid characteristics, while SBS latex-modified asphalts showed non-Newtonian behavior in the studied region of shear rates. In addition, it is clear that the type and composition of base asphalt had great influence on the flow behavior of SBS-latex-modified asphalt. The Newtonian fluid region of asphalt B was larger than that of asphalt B, which is attributed to the lower amount of asphaltenes in asphalt B. Meanwhile, it was found that the viscosity values of SBS-latex-modified asphalt B were all lower than that of modified asphalt A. That is to say, asphalt B with lower asphaltenes had the advantages of workability and inferior flow resistance ability.

In order to evaluate the effects of SBS latex dosage on the viscous properties of asphalt quantitatively, the relationship between shear rates and viscosity of asphalt can be fitted using Carreau model [8], which is as follows:

(1) $$\frac{\eta_{0}}{\eta }={\left[1+{\left(\frac{\dot{\gamma }}{{\dot{\gamma }}_{c}}\right)}^{2}\right]}^{s}$$

where η shows the viscosity of asphalt, η_0_ is the ultimate viscosity when the shear viscosity approaches zero which is also called the zero-shear viscosity (ZSV), s represents the slope of the fitting curve in the non-Newtonian region, while $\dot{\gamma }$ is shear rate, and $\dot{\gamma }$_c_ represents the critical shear rate value when the viscoelastic characteristic of asphalt binder changes from Newtonian behavior to the non-Newtonian region [40,41]. From Figure 6, the flow curve of asphalt fits the Carreau model fairly well, showing the parameters obtained from the Carreau model are very reliable. Table 3 shows the results of all the studied samples calculated from the Carreau model including zero-shear viscosity η_0_, critical shear rate value $\dot{\gamma }$_c_, and fitting curve slope s. It is clear that the neat asphalt has the lowest η_0_ value (ZSV), showing the poor flow resistance ability of the base asphalt at high temperature. From Table 3, it can be seen that the zero-shear viscosity of asphalt B was lower than that of asphalt A, while the $\dot{\gamma }$_c_ value of asphalt B was much higher than asphalt A. The results show that compared to asphalt A, asphalt B had a wider Newtonian fuild range and better workability, which is attributed to its higher content of light oil fractions and lower dosage asphaltenes. Meanwhile, it can also be found that the s value of asphalt B is higher than asphalt A, which means asphalt B has higher shear susceptibility than asphalt B, which is also connected to its chemical component characteristics.

Meanwhile, SBS latex can remarkably enhance the ZSV value of asphalt and this improvement was more obvious for modified asphalt A. Interestingly, the zero shear viscosity value of modified asphalt A was higher than that of modified asphalt B, indicating the viscous flow behavior was also significantly affected by the chemical composition of base asphalt. In terms of viscoelastic properties, high aromatic content and moderate asphaltenes dosage were beneficial for enhancing the flow resistance of asphalt. Furthermore, SBS particle-modified asphalt A had a higher η_0_ value than that of SBS-latex-modified asphalt A, even though the SBS latex content increased to 8 wt%, while the η_0_ value of SBS particle-modified asphalt B was lower than that of 6 wt% SBS particle-modified asphalt B. On the other hand, adding SBS latex results in a decrease in the critical shear rate $\dot{\gamma }$_c_ of the asphalt, indicating that the shear-thinning behavior of modified asphalt became more distinct. This is related to the presence of macro-molecules due to the incorporation of polymers and the complicated structure of modified asphalt being prone to disruption by shear stress. Moreover, the addition of SBS latex decreases the s value and improves the resistance to shear thinning of the modified asphalt.

**Figure 7:**
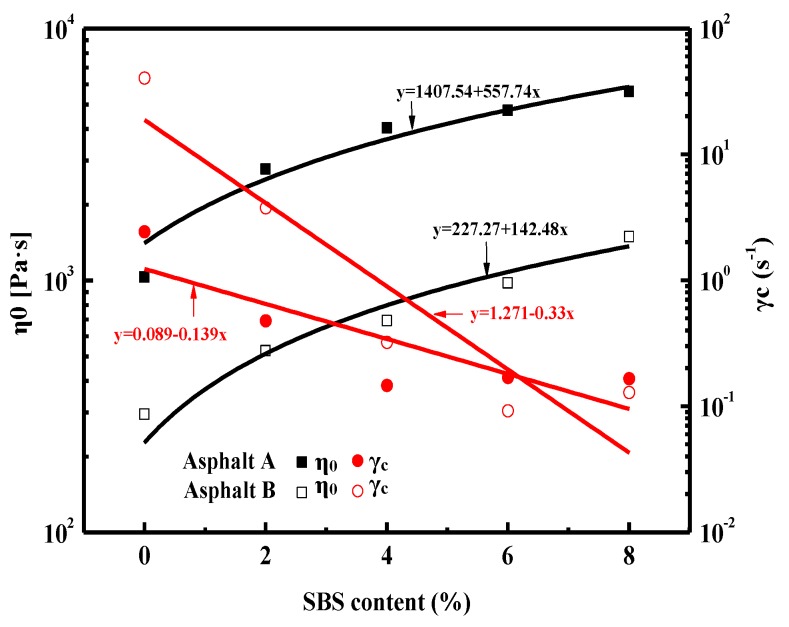
Effects of SBS latex content on the Carreau model parameters of modified asphalts.

The relationship between the SBS latex dosage and η_0_ value as well as the critical shear rate $\dot{\gamma }$_c_ was fitted by a linear formula as shown in Figure 7. With the increase in SBS latex, the η_0_ value of modified asphalt increased dramatically, while the critical shear rate $\dot{\gamma }$_c_ decreased, showing that SBS latex can improve the deformation resistance and shear-thinning behavior of asphalt. However, the influence of SBS latex content on the ZSV value of modified asphalt A was more obvious than that of modified asphalt B, while the effect on the $\dot{\gamma }$_c_ value of modified asphalt B was more distinct than that of modified asphalt B. Furthermore, the asphalt component can also affect the ZSV and $\dot{\gamma }$_c_ values of modified asphalt. It was found that the ZSV value of modified asphalt A was higher than that of modified asphalt B, while the $\dot{\gamma }$_c_ value of modified asphalt A was lower than that of modified asphalts B, indicating that asphalt A had better deformation resistance ability and was closer to the non-Newtonian fluid characteristic.

### 3.4. Creep and Recovery Behavior

**Figure 8:**
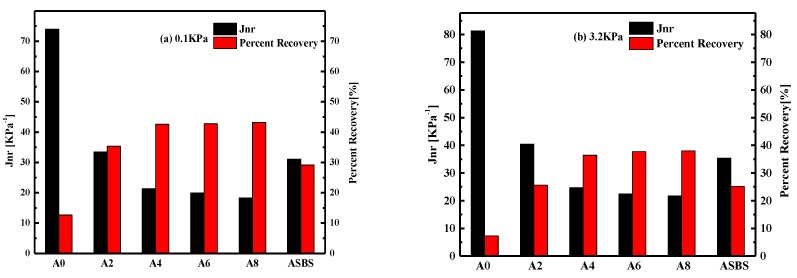

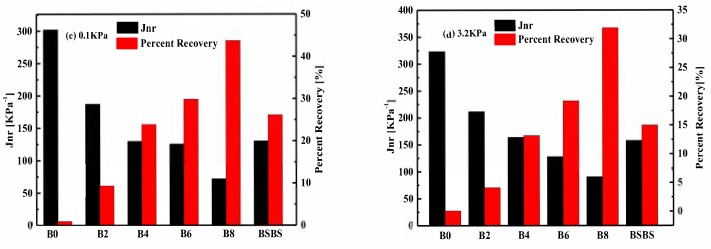
Effects of SBS latex content on the percent recovery (R%) and non-recovery compliance (Jnr) of modified asphalts.

In order to further evaluate the effects of SBS latex dosage on the high-temperature properties and recovery behavior of modified asphalt, MSCR tests were conducted with respect to AASHTO TP70 [25]. Figure 8 shows the MSCR results of asphalt samples at stress levels of 0.1 KPa and 3.2 KPa, respectively. It is well known that the percent recovery (R%) is an elastic response indicator of asphalt, and the increased value of R% indicates the improvement in the elastic component in asphalt. Meanwhile, non-recoverable creep compliance (Jnr) is well related to the rutting performance of asphalt, and the lower Jnr value represents the better resistance to permanent deformation of binder.

As expected, base asphalt had the lowest R% value and highest Jnr value regardless of the loading stress level. The R% value of asphalt was damaged when the loading stress increased from 0.1 KPa to 3.2 KPa, while the non-recoverable creep compliance Jnr increased dramatically. Meanwhile, adding SBS latex can remarkably increase the R% value and decrease the Jnr value of asphalt. The R% and Jnr value of modified asphalt increased and declined with the increase in SBS latex dosage, respectively. That is to say, SBS latex can improve the elastic recovery and deformation resistance of asphalt. However, the degree of influence of SBS latex content on the R% and Jnr value of modified asphalt depends on the chemical composition of the base asphalt. The effect of SBS latex dosage on the elastic properties of asphalt B was more obvious than that of asphalt A, which means the positive effect of SBS latex on the resistance to permanent deformation was more significant for asphalt B.

Additionally, modified asphalt A always had a higher R% and lower Jnr value than those of modified asphalt B, indicating modified asphalt A had better elastic response and deformation resistance performance than asphalt B. On the other hand, it was found that the R% value of SBS latex-modified asphalt was higher than that of SBS particle-modified asphalt, while the Jnr value of the former was lower than the latter, regardless of loading stress level when the SBS particle content was the same as the latex. Obviously, the SBS latex-modified asphalt possessed better elastic properties and anti-deformation abilities than conventional SBS particle-modified asphalt. As to asphalt A, both R% and Jnr values of SBS particle-modified asphalt were similar to that of 2 wt% SBS latex-modified asphalt, while SBS particle-modified asphalt B had the same grade of anti-rutting and elastic response performance with 4 wt% SBS latex-modified asphalt B. Furthermore, it can be found that the influence of SBS latex dosage on improving the permanent deformation resistance of asphalt A became less obvious when the SBS latex dosage exceeded to 4 wt%. However, the influence of SBS latex dosage on the recovery and creep compliance of asphalt B was more obvious. Therefore, asphalt B was more suitable to be modified with SBS latex which is consistent with the above results.

### 3.5. Compatibility 

As is well known, compatibility between polymer and base asphalt is very essential, which can result in phase separation and performance inhomogeneity of polymer-modified asphalt [12]. According to previous studies [9,22,42], a Cole-Cole diagram is the most efficient method to investigate the compatibility of polymer-modified asphalts. In this study, the Cole-Cole plot was selected to assess the compatibility differences during the preparation of the SBS latex-modified asphalts. It is well known that the compatibility of modified asphalt is related to the shape of Cole-Cole diagrams, and a symmetrical parabola shows great compatibility, while a conclusion of incompatibility between the asphalt and modifier is drawn when the shape deviates from symmetry [12].

**Figure 9:**
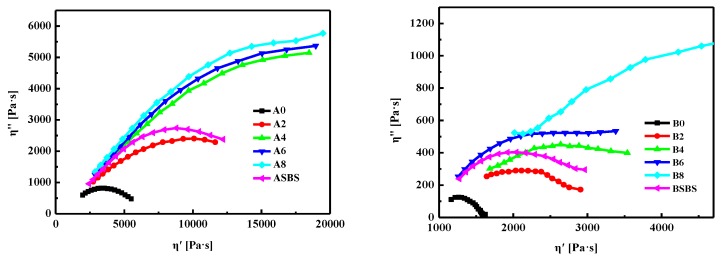
Cole-Cole plots of the base and SBS latex-modified asphalts.

Figure 9 displays the Cole-Cole diagrams of the base asphalt and the SBS latex-modified asphalts at 60 °C. For modified asphalt A, when the SBS latex dosage was lower than 4 wt%, the η” value first increased and then descended with the increase in η’. However, when the SBS latex dosage was larger than 4 wt%, the η” value of the modified asphalt rapidly increased with the increase in η’. This means that the compatibility of SBS latex-modified asphalt A became worse with the increase in the SBS latex dose; this is attributed to the concentretion limitation of resin in asphalt A. On the other hand, Cole-Cole plots of modified asphalt B present symmetrical parabolas when the SBS latex dosage was less than 8 wt%. With the increase in the SBS latex modifier, the shape of the Cole-Cole diagram deviated from this symmetry gradually, indicating the SBS latex dosage had a passive influence on the compatibility of modified asphalt.

Furthermore, the compatibility between asphalt and polymer also depended on the composition of the base asphalt. It was clear that the shape of the Cole-Cole plot for modified asphalt B was closer to symmetrical parabolas than that of modified asphalt A under the same SBS latex dosage [42,43,44]. This result showed that SBS latex-modified asphalt B had better compatibility than asphalt A, which is attributed to the composition differences of the base asphalt. From Table 1, it can be seen that asphalt B had more resins and a lower asphaltene concentration than asphalt A which is beneficial to the formation of a stable colloid structure and enhanced the compatibility between asphalt and SBS latex modifier. In conclusion, neat asphalt with a higher resins dosage and lower asphaltenes content is suitable to be used to prepare stable SBS latex-modified asphalt with satisfactory compatibility. Furthermore, it was found that the shape of the Cole-Cole plot for the SBS particle-modified asphalt trended to symmetrical parabolas better than that of the SBS latex-modified asphalt, regardless of the type and composition of the base asphalt. This indicates that the SBS latex-modified asphalt had worse compatibility than the conventional SBS particle-modified asphalt.

### 3.6. Storage Stability

**Figure 10:**
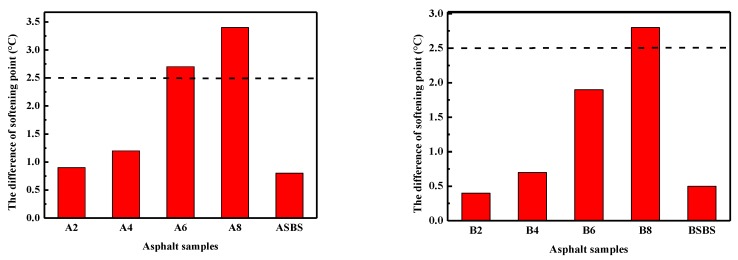
Effect of SBS latex content on the storage stability of modified asphalts.

Polymer-modified asphalts are multiphase systems and the difference in the solubility and density between polymer and asphalt can result in phase separation of the modified asphalt during high-temperature storage processes [45]. On the basis of this, a storage stability test was performed on SBS latex-modified asphalt according to the aforementioned testing method. To reveal the effect of SBS latex dosage on storage stability, thermal storage tests were conducted for all modified asphalts at 163 °C for 2 d. Figure 10 shows the results of storage stability tests for the SBS latex-modified asphalts. The results indicate that, with the increase in the SBS latex dosage, the softening point difference of the modified asphalts became gradually larger, showing that the higher SBS latex content had a passive influence on the storage stability and compatibility of modified asphalt. Meanwhile, the softening point difference value of modified asphalt B was lower than that of modified asphalt A. Hence, it was concluded that all SBS latex-modified asphalts suffered from phase separation during thermal storage, and the storage stability of modified asphalt was associated with the base asphalt composition as well as the SBS latex dosage.

It was found that the softening point difference of the modified asphalt increased as the SBS latex dosage increased. When the SBS dosage content reached 8 wt%, the softening point difference of modified asphalt A and B increased to 3.4 °C and 2.7 °C, respectively, which obviously exceeded the specification that polymer-modified asphalt is storage-stable with a softening point difference lower than 2.5 °C. These results demonstrate that a high SBS latex dosage has an adverse effect on the storage stability of modified asphalt. Furthermore, the thermal stability of modified asphalt also depends on the components of the base asphalt. It was clear that SBS latex-modified asphalt B had a smaller softening point difference value and greater storage stability than modified asphalt A which is associated with the asphalt components. From Table 1, asphalt A contained more asphaltenes and a lower amount of resins than asphalt B. Therefore, the difference in storage stability can be interpreted by the difference in asphalt compositions. Furthermore, the softening point difference of SBS latex-modified asphalt A was 1.9 °C larger than that of SBS particle-modified asphalt, while modified asphalt B had a 1.4 °C higher softening point difference. That is to say, SBS latex-modified asphalt had worse storage stability than conventional SBS particle-modified asphalt. Thus, it can be concluded that SBS latex-modified asphalt is suitable to be prepared and used at the construction site immediately. Certainly, SBS latex-modified asphalt with a lower content SBS latex can be prepared by the asphalt industry and transported to the site of the construction of an asphalt road.

## 4. Conclusions

This paper prepared and evaluated the rheological properties, compatibility, and storage stability of SBS latex-modified asphalt. The effects of SBS latex dosage and asphalt composition on the moduli, rutting and fatigue temperature, ZSV, R%, Jnr, compatibility, and storage stability of modified asphalt were investigated which were also compared with conventional SBS particle-modified asphalt. On the basis of the experimental results and discussion, the following conclusions were drawn:

(1) The SBS latex dosage had a great effect on the rheological properties of modified asphalt. With an increase in the SBS latex content, both the viscoelastic and high-temperature performance of modified asphalt improved including the moduli, rutting resistance, anti-fatigue, zero shear viscosity, and elastic recovery abilities.

(2) The chemical composition of neat asphalt also had a significant influence on the properties of SBS latex-modified asphalt. High asphaltenes and low resins are beneficial for anti-rutting and viscoelastic properties, while low asphaltenes and high resins are favorable to fatigue resistance ability, compatibility, and storage stability of modified asphalt.

(3) Compared to conventional SBS particle-modified asphalt, SBS latex-modified asphalt possesses higher moduli, greater rutting and fatigue resistance as well as superior viscoelastic performance which, however, has some disadvantages in terms of compatibility and storage stability.

(4) In this study, in comprehensively considering the balance of viscoelastic properties, compatibility, and storage stability of SBS latex-modified asphalt, the mixing content of SBS latex is recommended at 4.0 wt% which can be prepared at a construction site with a lower temperature. It is feasible for the SBS latex modifier to take the place of SBS particle and be used in the modification of asphalt.

## Figures and Tables

**Table 1:** Conventional properties and chemical compositions of base asphalt.

Test Properties	Asphalt A	Asphalt B	Methods
25 °C Penetration (0.1 mm)	68	71	ASTM D5 [18]
Softening point (°C)	49.8	48.6	ASTM D36 [19]
15 °C Ductility (cm)	>150	>150	ASTM D113 [20]
Saturates (wt%)	23.4	27.1	ASTM D4124 [21]
Aromatics (wt%)	41.2	32.9	
Resins (wt%)	26.8	39.1	
Asphaltenes (wt%)	8.6	0.9	
Colloidal index (CI) *	0.47	0.39	

* Colloidal index (CI) = (saturates + asphaltenes)/(aromatics + resins).

**Table 2:** Failure temperature and fatigue temperature of all asphalt samples.

SBS Latex Contents	Failure Temperature/°C		Fatigue Temperature/°C	
	Asphalt A	Asphalt B	Asphalt A	Asphalt B
0	78.13	68.12	10.12	4.79
2	83.61	74.35	9.45	1.80
4	86.52	77.85	7.33	−0.19
6	86.56	78.52	7.71	0.58
8	87.70	80.67	8.27	3.10
SBS particle	83.89	72.37	7.74	2.16

**Table 3:** The results calculated from the Carreau model at 60 °C of all studied samples.

Items	SBS Latex Contents/wt%					
	0	2	4	6	8	SBS Particle
Asphalt A	A0	A2	A4	A6	A8	ASBS
η_0_ × 10^−3^ (Pa·s)	1.032	2.773	4.733	5.026	5.629	5.718
$\dot{\gamma }$_c_ (s^−1^)	2.416	0.471	0.142	0.169	0.165	0.155
s	0.300	0.193	0.146	0.149	0.128	0.126
Asphalt B	B0	B2	B4	B6	B8	BSBS
η_0_ × 10^−3^ (Pa·s)	0.295	0.527	0.692	0.977	1.494	0.762
$\dot{\gamma }$_c_ (s^−1^)	40.207	3.743	0.321	0.092	0.128	0.164
s	1.141	0.206	0.082	0.089	1.107	0.090
